# Complete Mitochondrial Genome Sequence of *Colletotrichum siamense* Isolated in South Korea

**DOI:** 10.1128/mra.01055-21

**Published:** 2022-04-26

**Authors:** Sung-Eun Cho, Ji Yeon Oh, Dong-Hyeon Lee

**Affiliations:** a Forest Biodiversity Division, Korea National Arboretum, Pocheon, South Korea; b Division of Forest Insect Pests and Diseases, National Institute of Forest Science, Seoul, South Korea; University of California, Riverside

## Abstract

The complete mitochondrial genome of *Colletotrichum siamense* is characterized. The circular genome has a size of 52,710 bp, with a GC content of 34.45%, and contains 15 protein-coding genes, 23 tRNA genes, and 2 rRNA genes.

## ANNOUNCEMENT

Among the top 10 fungal pathogens of scientific and economic importance, the genus *Colletotrichum* Corda 1831 is considered the eighth most important phytopathogenic fungus (1); it causes typical symptoms of anthracnose on fruit, vegetable, and ornamental hosts worldwide, leading to severe losses in the yield and quality of hosts (2).

In 2020, severely infected fruit of pecan, Carya illinoinensis (Wangenh.) K. Koch, showing distinct anthracnose symptoms was observed in pecan orchards in Uiseong (36°21′31.5″N, 128°27′15.9″E) and Miryang (35°22′54.9″N, 128°48′06.5″E) in South Korea, and the causal agent of anthracnose on pecan was identified as Colletotrichum siamense Prihastuti, L. Cai & K. D. Hyde 2009 (3).

The Korean isolate of C. siamense, which was deposited in the Korean Agricultural Culture Collection (KACC), National Institute of Agricultural Sciences, South Korea (http://genebank.rda.go.kr) (accession no. KACC 49782), was obtained from the culture collection (Cultures of Dong-Hyeon) of the National Institute of Forest Science, South Korea (https://nifos.forest.go.kr) (accession no. CDH2020-20) (3). Genomic DNA was extracted from the mycelium using Maxwell 16 DNA purification kits (Promega, USA). An Illumina paired-end (PE) library was constructed and sequenced using the Illumina HiSeq X platform with 151-bp PE reads. Raw sequencing data (2.2 Gb) were trimmed using the quality_trim program in the CLC Assembly Cell package v. 4.2.1 (Qiagen, Denmark) with default parameters (Phred scores of >20) and then were used for *de novo* assembly of the mitochondrial genome as in a previous study (4). In brief, trimmed high-quality read sequences were *de novo* assembled using the clc_novo_assemble program in CLC Assembly Cell with default parameters, and then mitochondrial contigs were selected and ordered by similarity searches with mitochondrial sequences from NCBI Organelle Genome Resources (https://www.ncbi.nlm.nih.gov/genome/organelle). The selected contigs were merged and generated a draft mitochondrial genome sequence. Subsequently, gaps were filled and errors were corrected by PE read mapping and manual curation as in a previous study (5). The final complete mitochondrial genome sequence was annotated using the GeSeq (6) and Artemis (7) programs with mitochondrial reference genomes (GenBank accession no. KX885100, KX885103, KX885101, KX034082, and KX885105). In addition, the precise gene regions were determined by manual curation based on BLAST searches against the mitochondrial reference genomes.

The mitochondrial genome of the Korean isolate of C. siamense is a circular molecule of 52,710 bp, with a GC content of 34.45%. Gene prediction and annotation identified 15 protein-coding genes, 23 tRNA genes, and 2 rRNA genes in the genome. Comparative analysis with the mitochondrial genomes of Chinese isolates of C. siamense (GenBank accession no. KX885098 to KX885103) (8), based on sequence alignment using BLAST and MAFFT (http://mafft.cbrc.jp/alignment/server/index.html), revealed that the genome of the Korean isolate showed 99.2 to 99.9% similarity to the Chinese isolates and had 35 polymorphic sites, including 15 single-nucleotide polymorphisms (SNPs) and 20 indels. These findings indicated that the Korean isolate of C. siamense is distinct from the Chinese isolates.

**FIG 1:**
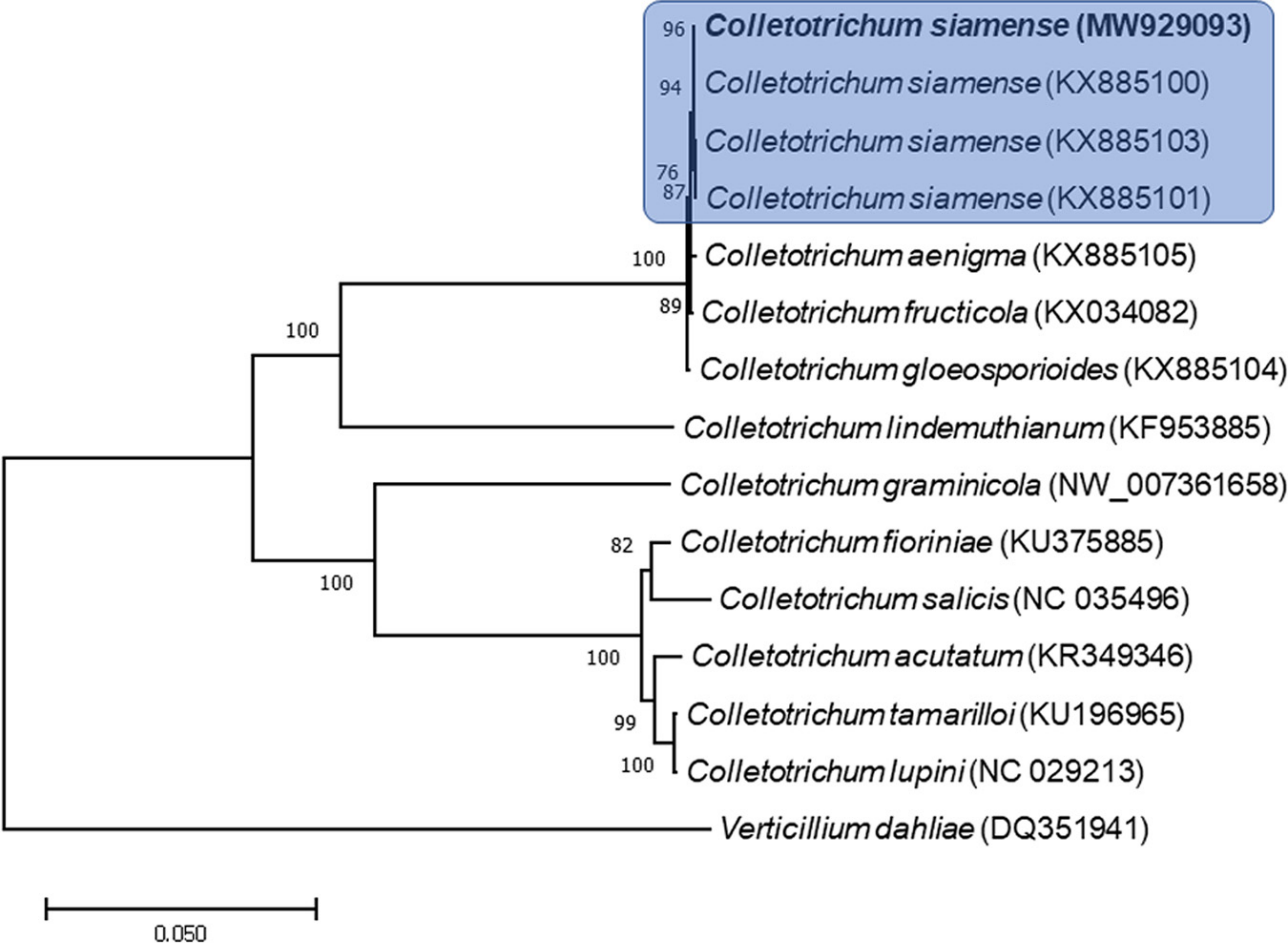
ML phylogenetic tree of the mitochondrial genomes of the *Colletotrichum siamense* Korean isolate and related species. Eleven protein-coding sequences, including *atp8*, *atp9*, *cox2*, *nad5*, *cob*, *atp6*, *nad1*, *cox1*, *rps3*, *nad6*, and *cox3*, that were conserved in the mitochondrial genomes of 15 species underwent multiple alignment using MAFFT (http://mafft.cbrc.jp/alignment/server/index.html) and were used to generate the phylogenetic tree with MEGA v. 7.0 (9). The bootstrap support values (>50%) from 1,000 replicates are indicated on the nodes. The Korean isolate, Colletotrichum siamense KACC 49782, obtained and used in the phylogenetic analysis is represented in bold. The GenBank accession no. of mitochondrial genome sequences used for this tree are indicated in parentheses.

Phylogenetic analysis using the Korean isolate of C. siamense with other taxa was performed using a maximum likelihood (ML) method with conserved protein-coding sequences and revealed that the Korean isolate is located in the same clade as other *Colletotrichum* strains and close associates (Fig. 1).

### Data availability.

The genome sequence data that support the findings of this study are available in NCBI GenBank under the accession no. MW929093. The associated BioProject, BioSample, and SRA no. are PRJNA722052, SAMN18744303, and SRR14241033, respectively.
